# Psychometric Evaluation of the Romanian Quality of Life in Adult Cancer Survivors (QLACS) in Women with Breast Cancer: A Cross-Sectional Study

**DOI:** 10.3390/jcm15187151

**Published:** 2026-09-15

**Authors:** Paula Alexandra Blanaru, Elena Porumb-Andrese, Cristian Mârtu, Ramona Gabriela Ursu, Monica Mihaela Scutariu, Gabriela Rusu Zota, Vlad Porumb

**Affiliations:** 1 Grigore T. Popa University of Medicine and Pharmacy Iasi, 700115 Iasi, Romania; paula-alexandra.blanaru@d.umfiasi.ro (P.A.B.); martu.cristian@umfiasi.ro (C.M.); mihaela.scutariu@umfiasi.ro (M.M.S.); rusu.i.gabriela@umfiasi.ro (G.R.Z.); vlad.porumb@umfiasi.ro (V.P.); 2 Onești Municipal Hospital, 601048 Onesti, Romania; 3 Clinical Railways Hospital, 700115 Iasi, Romania; 4 Dr. Iacob Czihac Emergency Military Clinical Hospital, 700115 Iasi, Romania

**Keywords:** quality of life, patient-reported outcome measures, psychometrics, patient outcome assessment, neoplasms

## Abstract

The increase in the number of people living after an oncological diagnosis requires the use of instruments capable of assessing both general dimensions and specific aspects of quality of life. The study aimed to evaluate the psychometric properties of the Romanian version of the Quality of Life in Adult Cancer Survivors (QLACS) questionnaire, analyze the relationships between its scores, age, and residence environment, and identify distinct quality of life profiles. **Methods**: A cross-sectional study was conducted in 224 adult women with a confirmed diagnosis of breast cancer. The internal consistency of the 12 QLACS domains was assessed by Cronbach’s alpha coefficient. The structure of the instrument was explored by principal component analysis with Oblimin rotation. Differences according to residence were analyzed by the Mann–Whitney test, and the relationships with age by the Spearman coefficient. Multivariable regression models including age and residential environment were fitted for all 12 QLACS domains. Quality of life profiles were identified by hierarchical classification and k-means analysis. **Results**: Cronbach’s alpha coefficients ranged from 0.832 to 0.925, indicating good internal consistency across the 12 QLACS domains. Principal component analysis retained 11 components, explaining 73.738% of the total variance, providing preliminary exploratory support for the multidimensional structure of the instrument. Most domains were clearly delineated, whereas Cognitive problems partially overlapped with Recurrence distress. Urban residence was associated with higher Cognitive problems scores (r = 0.17), whereas rural residence was associated with higher Family distress scores (r = 0.29). Age was negatively correlated with Negative feelings (rho =−0.200, *p* = 0.003). A weak exploratory association was also observed with Recurrence distress (rho = 0.135, *p* = 0.043), although the corresponding multivariable model was not statistically significant. Cluster analysis identified three exploratory quality-of-life profiles, and residential environment was associated with cluster membership, although the association was small (Cramer’s V = 0.188). **Conclusions**: The Romanian QLACS showed good internal consistency and preliminary exploratory structural evidence in women with breast cancer. Further studies are needed to confirm its psychometric properties.

## 1. Introduction

The number of people living with cancer is increasing worldwide. GLOBOCAN estimates for 2022 indicate nearly 20 million new cases and approximately 9.7 million cancer deaths. If current trends of population growth and aging continue, the annual number of new cases could reach approximately 35 million by 2050, a 77% increase over 2022 [1]. In the United States, where survival data are systematically tracked, approximately 18.6 million people were living with a previous cancer diagnosis as of 1 January 2025, and their number is expected to exceed 22 million by 2035 [2].

These data reflect a significant shift in oncology. More people are living years or even decades after diagnosis, sometimes with lingering effects of the disease and treatment. Consequently, the evaluation of oncological outcomes can no longer be limited to disease control and survival. It also becomes necessary to assess how cancer influences physical functioning, emotional and cognitive state, social relationships and family life.

Cancer survivorship represents a continuum extending from diagnosis through treatment and post-treatment follow-up. QLACS was originally developed for long-term cancer survivors, but subsequent studies have also evaluated the instrument in shorter-term and early post-treatment survivors [3,4,5,6].

Quality of life is a multidimensional construct that includes physical, emotional, cognitive and social functioning, as well as the individual’s perception of health and ability to perform usual activities [7]. In cancer survivors, several of these dimensions may remain affected after diagnosis and treatment. Persistent fatigue, pain, cognitive difficulties, emotional distress, sexual problems, and changes in body image have all been reported and may interfere with daily functioning and social participation [8,9,10,11,12,13,14,15]. These problems may coexist and influence one another, supporting the need for instruments that assess several quality-of-life domains rather than isolated symptoms.

Cancer-related concerns may also persist beyond the period of active treatment. Fear of recurrence, worries about family cancer risk and genetic predisposition, as well as changes in priorities and perceived positive consequences of the cancer experience, may all contribute to quality of life [16,17,18,19,20]. In addition, place of residence may influence access to oncology and supportive-care services. Rural patients may face longer travel distances, higher indirect costs, and more limited access to specialized care, although rural–urban differences are not consistent across all quality-of-life domains [21,22,23].

The Quality of Life in Adult Cancer Survivors (QLACS) questionnaire was specifically developed to assess multidimensional quality of life in adult cancer survivors. Unlike instruments focused primarily on general health status or on symptoms during active cancer treatment, QLACS includes both generic and cancer-specific domains, allowing the simultaneous assessment of physical, emotional, cognitive, sexual, family, financial, appearance-related, and recurrence-related concerns. The original instrument demonstrated good internal consistency and supported a multidimensional structure, and subsequent studies evaluated its psychometric performance in long-term breast cancer survivors, shorter-term survivors, and women in the early post-treatment period [3,4,5,6]. A Spanish cross-cultural adaptation also reported satisfactory reliability and validity [24]. These studies support the use of QLACS in different survivorship settings, but also indicate the importance of examining whether its psychometric structure is maintained when the instrument is adapted to another language and cultural context.

In Romania, approximately 100,471 new cancer cases and 45,866 cancer-related deaths were estimated for 2022. Although cancer incidence remains slightly below the European Union average, mortality is high, while the increasing prevalence indicates a growing population requiring long-term follow-up and supportive care [25,26]. Access to oncology services and post-treatment monitoring remains uneven across regions and between urban and rural areas, and limitations have also been reported in the collection of oncology data and patient-reported outcomes [25,26].

In Romania, quality of life in cancer patients has mainly been evaluated using instruments such as the EORTC QLQ-C30, FACT-B, and SF-36 [27,28]. Although these questionnaires are useful for assessing general functioning, symptoms, and disease-related quality of life, QLACS focuses more specifically on the survivorship period. It includes both general domains and cancer-related concerns, such as Recurrence distress, Family distress, financial difficulties, appearance concerns, and perceived benefits of the cancer experience. QLACS has not yet been psychometrically evaluated in Romanian. It is therefore important to examine whether its multidimensional structure and internal consistency are also maintained in Romanian women with breast cancer. In addition, the relationships of these dimensions with age and residential environment have been insufficiently studied in women with breast cancer. It is also unclear whether subgroups with different quality of life profiles can be identified in this population.

Previous studies using cluster or latent profile analysis have identified different subgroups of cancer survivors based on combinations of symptoms, psychosocial difficulties, and quality-of-life characteristics [29,30,31,32,33,34]. Similar approaches have also been used in breast cancer survivors. In this context, cluster analysis may help explore whether participants with different combinations of QLACS domain scores can be identified.

The present study aimed to evaluate the psychometric properties of the Romanian version of the QLACS questionnaire in adult women with a confirmed diagnosis of breast cancer. Internal consistency and the instrument’s exploratory structure were examined, along with the distribution of domain scores. Associations between QLACS scores, age, and residential environment were also analyzed, and cluster analysis was used to explore distinct quality-of-life profiles.

It was hypothesized that the Romanian version of the QLACS would show adequate internal consistency and a multidimensional structure broadly consistent with the original instrument. It was also assumed that age and residential environment would be differentially associated with certain quality of life domains. Finally, based on previous evidence of heterogeneity among cancer survivors, we explored whether participants could be grouped into distinct quality-of-life profiles based on combinations of QLACS domain scores.

## 2. Materials and Methods

### 2.1. Study Design and Ethical Issues

A cross-sectional observational study was conducted to translate, preliminarily adapt, and evaluate the psychometric properties of the Romanian version of the Quality of Life in Adult Cancer Survivors (QLACS) questionnaire (Department of Public Health Sciences, Wake Forest University School of Medicine, Medical Center Boulevard, Winston-Salem, NC 27157, USA). Data was collected once from each participant by individually completing the questionnaire.

The study was conducted during May–June 2026, within the “Saint Hierarch Dr. Luca” Municipal Hospital, Onești, Romania. The research protocol was approved by the Institutional Ethics Committee (No. 1754/20 May 2026). The study was conducted in compliance with the principles of the Declaration of Helsinki.

Before inclusion in the study, the purpose of the research, how to complete the questionnaire, and the voluntary nature of participation were explained to the participants. All participants signed the informed consent. The data were collected and analyzed confidentially, without recording any information that would allow direct identification of the participants.

### 2.2. Participants and Selection Criteria

The study included 224 adult women, all with a confirmed diagnosis of breast cancer. Participants were recruited through convenience sampling from patients evaluated at the “Saint Hierarch Dr. Luca” Municipal Hospital, Onești, during the study period.

In the present study, the term “cancer survivor” is used in accordance with the broad survivorship concept underlying the QLACS instrument, referring to individuals living after a cancer diagnosis. However, QLACS was originally developed for adult cancer survivors; detailed information regarding time since diagnosis, current treatment status, and time since treatment completion was not collected in the present study. Therefore, participants could not be classified according to specific phases of cancer survivorship.

Detailed clinical information regarding breast cancer stage, treatment modalities, recurrence or metastatic status, and current treatment was not collected. Therefore, the study population could not be further characterized according to these clinical variables.

The final sample consisted of 224 participants for the 47 QLACS items, corresponding to a participant-to-item ratio of approximately 4.8:1. Sample adequacy was not considered on the basis of this ratio alone, as recommendations for psychometric analyses also take into account the characteristics of the data and the resulting factor solution [35,36]. The achieved sample was considered acceptable for an exploratory analysis, although it remains relatively modest for a 47-item questionnaire. Therefore, the identified structure should be confirmed in a larger independent sample.

The inclusion criteria were age of at least 18 years, a confirmed diagnosis of breast cancer, knowledge of the Romanian language, ability to understand and independently complete the questionnaire, and expression of informed consent for participation.

Patients with severe cognitive, neurological, or psychiatric disorders that could affect the understanding of the items, those who could not independently complete the questionnaire, and cases in which the instrument was partially or inadequately completed were excluded. Only fully completed questionnaires were included in the final analysis.

### 2.3. Research Instrument

The Quality of Life in Adult Cancer Survivors (QLACS) questionnaire was developed to assess the quality of life in adults with a history of cancer. The instrument contains 47 items organized into 12 domains, of which seven are general, and five are specific to the cancer experience [3,4].

A. Generic domains

Positive feelings assess the presence of positive affective states and include the following items:
  Item 6: You felt happy.
  Item 8: You enjoyed life.
  Item 22: You had a positive outlook on life.
  Item 28: You were content with your life.

Fatigue/Energy assesses energy levels and the presence of fatigue:
  Item 1: You had the energy to do the things you wanted to do.
  Item 5: You felt fatigued.
  Item 11: You did not have energy to do the things you wanted to do.
  Item 14: You felt tired a lot.

Social avoidance aims to avoid relationships and social activities:
  Item 15: You were reluctant to start new relationships.
  Item 18: You avoided social gatherings.
  Item 20: You avoided your friends.
  Item 25: You were reluctant to meet new people.

Pain evaluates the presence of pain and its influence on activities and emotional state:
  Item 13: You were bothered by pain that kept you from doing the things you wanted to do.
  Item 17: Your mood was disrupted by pain or its treatment.
  Item 21: You had aches or pains.
  Item 27: Pain or its treatment interferes with your social activities.

Negative feelings evaluate depressive mood, anxiety, and emotional instability:
  Item 7: You felt blue or depressed.
  Item 9: You worried about little things.
  Item 19: You were bothered by mood swings.
  Item 24: You felt anxious.

Cognitive problems refer to difficulties in attention, memory, and concentration:
  Item 2: You had difficulty doing activities that require concentration.
  Item 3: You were bothered by having a short attention span.
  Item 4: You had trouble remembering things.
  Item 23: You were bothered by forgetting what you started to do.

Sexual problems evaluate sexual interest, sexual functioning, and satisfaction with sexual life:
  Item 10: You were bothered by being unable to function sexually.
  Item 12: You were dissatisfied with your sex life.
  Item 16: You lacked interest in sex.
  Item 26: You avoided sexual activity.

B. Cancer-specific domains

Financial problems: Evaluate financial difficulties associated with oncological diagnosis and treatment:
  Item 30: You had financial problems because of the cost of cancer surgery or treatment.
  Item 37: You had problems with insurance because of cancer.
  Item 43: You had money problems that arose because you had cancer.
  Item 45: You had financial problems due to a loss of income because of cancer.

Cancer benefits evaluate positive changes perceived because of the oncological experience:
  Item 29: You appreciated life more because of having had cancer.
  Item 32: You realized that having had cancer helps you cope better with problems now.
  Item 40: You felt that cancer helped you to recognize what is important in life.
  Item 41: You felt better able to deal with stress because of having had cancer.

Family distress evaluates concerns regarding the oncological risk of family members:
  Item 31: You worried that your family members were at risk of getting cancer.
  Item 34: You worried about whether your family members might have cancer-causing genes.
  Item 42: You worried about whether your family members should have genetic tests for cancer.

Appearance concerns evaluate concerns related to changes in physical appearance caused by the disease or treatment:
  Item 33: You were self-conscious about the way you look because of your cancer or its treatment.
  Item 35: You felt unattractive because of your cancer or its treatment.
  Item 38: You were bothered by hair loss from cancer treatment.
  Item 44: You felt people treated you differently because of changes to your appearance due to your cancer or its treatment.

Recurrence distress evaluates the fear of relapses and concerns related to the evolution of the disease:
  Item 36: You worried about dying from cancer.
  Item 39: You worried about cancer coming back.
  Item 46: Whenever you felt a pain, you worry that it might be cancer again.
  Item 47: You were preoccupied with concerns about cancer.

Each item is rated on a seven-point frequency scale, from 1, corresponding to the answer “never”, to 7, corresponding to the answer “always”. Participants were asked to indicate how often each statement was true in the last four weeks.

The score for each domain was calculated as the mean of its constituent items, with possible values ranging from 1 to 7. Positively worded items within negatively oriented domains were reverse-coded before domain scores were calculated, including the positive energy item in the Fatigue/Energy domain. The Positive feelings domain was analyzed in its original direction, with higher scores indicating more positive feelings. The Family distress score was calculated as the mean of its three items and was not rescaled, because mean scoring preserves the same 1–7 scale across domains regardless of the number of items. For the negatively oriented domains, higher scores indicate greater difficulties, whereas higher scores for Positive feelings and Cancer benefits indicate a more favorable experience. The domains were analyzed separately, without calculating an overall QLACS score.

### 2.4. Translation and Preliminary Linguistic Adaptation of the QLACS Questionnaire

The original English version of the QLACS questionnaire was independently translated into Romanian by two investigators [C.B. and B.L.]. The two versions were compared, and differences in terminology and wording were discussed until a common version was obtained. In choosing the wording, the aim was to preserve the conceptual meaning of the original items and to use a clear and appropriate language for Romanian-speaking patients.

The clarity and ease of understanding of the translated version were checked in the initial stage of administration, on the first 100 patients recruited. They were asked to report any difficulties in understanding the instructions, items, or response options. No wording was identified that would require modification of the meaning of the items, the structure of the instrument, or the response scale, so the same version of the questionnaire was used for all participants.

The 100 patients involved in the clarity check were part of the final batch. Because the instrument was not modified after this stage, their responses were retained and included in the psychometric analysis performed on the entire sample of 224 participants. The adaptation process did not change the number of items, response options, or domain structure of the original instrument.

No back-translation, independent bilingual expert review, formal expert committee assessment, or formal content validity evaluation was performed. Therefore, the procedure should be considered a preliminary linguistic adaptation rather than a comprehensive cross-cultural validation.

### 2.5. Statistical Analysis and Psychometric Validation

The data were processed using IBM SPSS Statistics, version 26.0 (IBM Corp., Armonk, NY, USA). The evaluation of the Romanian version of the QLACS questionnaire included descriptive analysis of items and domain scores, assessment of internal consistency, examination of factor structure, and analysis of relationships between QLACS scores and participant characteristics.

Categorical variables were presented as absolute frequencies and percentages. For continuous variables, mean, standard deviation, median, and minimum and maximum values were calculated. For the scores of the 12 QLACS domains, skewness and kurtosis values were also calculated to describe the shape of the distributions.

#### 2.5.1. Internal Consistency

The internal consistency of each domain was assessed using Cronbach’s alpha coefficient. The value of the alpha coefficient was also calculated in the case of successive elimination of each item, to determine whether its exclusion would have improved the internal consistency of the domain.

#### 2.5.2. Factor Structure Analysis

Before the factor analysis, the adequacy of the data was verified by the Kaiser–Meyer–Olkin index and the Bartlett test of sphericity. The commonalities after extraction were also analyzed to assess the proportion of the variation in each item explained by the solution obtained.

The structure of the instrument was explored using principal component analysis (PCA) with Oblimin oblique rotation. PCA was selected as an exploratory approach to examine the empirical grouping of items and its correspondence with the theoretical QLACS domains. Because PCA does not estimate latent factors in the same way as common factor analysis, the findings were interpreted as exploratory structural evidence rather than as confirmation of factorial validity. The Kaiser criterion was used to retain components with eigenvalues greater than 1.

The interpretation of the solution was based on the pattern matrix. The magnitude of the factor loadings, the grouping of items corresponding to the same domain, and possible loadings on several components were monitored. Factor loadings with values of at least 0.40 were considered. The solution obtained was compared with the theoretical structure of the original instrument, consisting of 12 domains.

#### 2.5.3. Comparative and Correlational Analyses

The normality of the distribution of QLACS scores was verified by the Kolmogorov–Smirnov, Lilliefors correction, and Shapiro–Wilk tests. Since the distributions of domain scores deviated significantly from the normal distribution, nonparametric tests were used for unadjusted comparisons and correlation analyses.

Differences between rural and urban participants were assessed by the Mann–Whitney U test. The effect size was calculated with the formula:

r = |Z|/√N,

where Z represents the standardized test value and N the total number of participants included in the comparison.

The association between age and scores for each QLACS domain was analyzed using the Spearman correlation coefficient.

Analyses of associations between QLACS domains, age, and residential environment were considered exploratory, and no adjustment for multiple comparisons was applied to these analyses.

#### 2.5.4. Regression Models

Age and residential environment were included a priori as predictors in the multivariable models, because both variables were of interest according to the study objectives. This approach avoided selecting predictors solely based on statistical significance in preliminary univariate analyses. Age and residential environment were included simultaneously in each model to estimate the association of each predictor with the domain score after adjustment for the other variable.

Residential environment was coded with value 1 for rural area and with value 2 for urban area. For each model, the unstandardized regression coefficient, standard error, standardized coefficient, 95% confidence interval, *p*-value, R^2^ coefficient of determination, and adjusted R^2^ value were reported.

Multicollinearity between predictors was assessed by the variance inflation factor. Linearity of relationships, distribution of residuals, homoscedasticity, and presence of influential cases were verified by examining residual plots and diagnostic indicators of the models.

Missing data imputation was not necessary, as only fully completed questionnaires were included in the final analysis.

### 2.6. Cluster Analysis

To identify distinct quality of life profiles, the standardized scores of the 12 QLACS domains were used. Standardization was performed before classification to avoid the dispersion differences between domains that would disproportionately influence the formation of clusters.

In the first step, hierarchical clustering was performed using Ward’s method. The dendrogram and changes in the agglomeration process were examined to identify a plausible number of clusters. This exploratory assessment suggested a three-cluster solution, which was subsequently used in the k-means analysis. No additional internal validation procedure, such as silhouette analysis or bootstrap stability assessment, was performed.

Subsequently, the k-means method was applied, with a three-cluster solution previously established. The analysis was performed based on the standardized scores of all 12 QLACS domains.

Differences between clusters were assessed using the Kruskal–Wallis test. For domains where the global test was significant, post hoc comparisons were performed between pairs of clusters, with Bonferroni adjustment of *p*-values.

Age differences between the three clusters were analyzed using the Kruskal–Wallis test. The association between cluster membership and residential environment was assessed using the chi-square test, and the strength of the association was estimated using Cramer’s V coefficient.

All statistical tests were two-sided, and the threshold for statistical significance was set at *p* < 0.05. Bonferroni-adjusted *p*-values were used for post hoc comparisons between clusters. Analyses examining associations between QLACS domains, age, and residential environment were considered exploratory, and no adjustment for multiple testing was applied to these analyses. Therefore, individual *p*-values, particularly those close to the significance threshold, should be interpreted cautiously.

## 3. Results

### 3.1. General Characteristics of the Study Group

The study included 224 participants, with confirmed diagnosis of breast cancer. All participants were female. The mean age was 50.84 years, with values between 23 and 81 years, which shows a wide age distribution in the group. Most participants came from urban areas (61.6%), and 38.4% from rural areas (Table 1).

### 3.2. Psychometric Validation of the Quality of Life in Adult Cancer Survivors (QLACS) Questionnaire

The internal consistency analysis showed that all the questionnaire domains presented high values of the Cronbach’s Alpha coefficient, ranging between 0.832 and 0.925. The highest values were obtained for the domain Negative feelings (α = 0.925), followed by Positive feelings (α = 0.899) and Cancer benefits (α = 0.893), which suggests a very good internal consistency of the items that compose these dimensions. Similar values were also observed for the domains Recurrence distress (α = 0.884), Appearance concerns (α = 0.880), Fatigue/Energy (α = 0.872), Financial problems (α = 0.870), and Pain (α = 0.866). The domains Sexual problems (α = 0.857), Family distress (α = 0.855), Social avoidance (α = 0.852), and Cognitive problems (α = 0.832) also had values indicating good internal consistency. Although the domain Cognitive problems recorded the lowest value of all the domains analyzed, the coefficient obtained remains above the usually accepted thresholds for the internal reliability of a scale. In the case of the Family distress domain, the value α = 0.855 is particularly relevant because the domain includes only three items, indicating good internal consistency despite the domain containing only three items (Table 2).

Overall, the results indicate good internal consistency across the QLACS domains. However, Cronbach’s alpha alone does not establish unidimensionality or construct validity.

Item-level descriptive statistics are presented in Supplementary Table S1. Item means ranged from 2.08 to 6.08, and standard deviations ranged from 0.742 to 1.600. The lowest means were recorded for the domain Financial problems, with values between 2.08 and 2.18. At the opposite end, the highest means were observed for the domain Cognitive problems, where values ranged from 5.79 to 6.08. High means were also observed for the domains Appearance concerns and Recurrence distress.

The Cronbach’s alpha if item deleted values ranged from 0.773 to 0.913. For each domain, these values were lower than the Cronbach’s alpha coefficient calculated for the respective domain, which shows that the removal of any item would not have improved internal consistency. The highest value was observed in the domain Negative feelings (0.913), and the lowest in the domain Cognitive problems (0.773). No item deletion would have improved the Cronbach’s alpha of its corresponding domain.

Before factor analysis, the adequacy of the data was checked. The Kaiser–Meyer–Olkin index had a value of 0.877, indicating good adequacy of the sample for this type of analysis. The Bartlett test of sphericity was statistically significant, χ^2^(1081) = 6859.669, *p* < 0.001, which shows that the correlation matrix is not an identity matrix (Table 3). Based on these results, the questionnaire items were considered suitable for factor analysis.

The results regarding the communalities are presented in Supplementary Table S2. The communalities after extraction ranged between 0.683 and 0.878, with all values being above the threshold of 0.50. This result indicates that the items were adequately represented in the extracted solution. The lowest value was recorded for the item “You were bothered by having a short attention span” (0.595), and the highest for the item “You felt happy” (0.878).

The results regarding the total variance explained are presented in Supplementary Table S3. The principal components analysis retained 11 components with eigenvalues greater than 1, which together explained 73.738% of the total variance. The highest contributions were observed for the first three components, with percentages of 15.062%, 10.657%, and 8.492%. For the other components retained, the percentage of explained variance was lower, ranging between 7.510% and 2.739%.

The following component had an eigenvalue of 0.859, below the threshold of 1, and was not included in the final solution. The 11-component solution consisted of 10 components corresponding to distinct theoretical QLACS domains and one component combining Recurrence distress items with three Cognitive problems items. The domains Negative feelings, Pain, Cancer benefits, Family distress, Social avoidance, Fatigue/Energy, Appearance concerns, Positive feelings, Financial problems, and Sexual problems each formed distinct components. The Cognitive problems domain did not emerge as a separate component. Three of its four items loaded on the same component as Recurrence distress, with relatively modest loadings ranging from 0.402 to 0.446, while the item “You had trouble remembering things” did not reach the predefined loading threshold of 0.40. Thus, the 11-component solution differed from the theoretical 12-domain structure mainly through the absence of a distinct Cognitive problems component.

The pattern matrix after the Oblimin rotation is presented in Supplementary Table S4. For most domains, the items clearly clustered on the extracted components, with high factor loadings. The domains Negative feelings, Pain, Cancer benefits, Family distress, Social avoidance, Fatigue/Energy, Appearance concerns, Positive feelings, Financial problems, and Sexual problems were represented by distinct components.

High loadings were observed for Negative feelings (0.833–0.872), Pain (0.800–0.859), Cancer benefits (0.799–0.895), Positive feelings (0.823–0.883), Appearance concerns (0.818–0.884), Financial problems (0.764–0.892), and Sexual problems (0.750–0.852). These values indicate a good association between the items and the corresponding components.

Three cognitive items loaded on the same component as the items in Recurrence distress, with values between 0.402 and 0.446. The item “You had trouble remembering things” did not exceed the threshold for displaying factor loadings. This overlap may explain the extraction of 11 components, although the theoretical structure of the instrument includes 12 domains. Overall, the results support the multidimensional nature of the questionnaire. However, the Cognitive problems domain should be interpreted with caution, given the lower loadings and partial overlap with the Recurrence distress domain.

### 3.3. Descriptive Statistics of QLACS Domain Scores

Descriptive statistics of QLACS domain scores are presented in Table 4. The highest mean scores were obtained for Cognitive problems (5.92 ± 0.79), Appearance concerns (5.57 ± 1.19), Pain (5.29 ± 0.72), Recurrence distress (5.13 ± 1.03), and Cancer benefits (5.11 ± 1.28). In contrast, the lowest means were recorded for Financial problems (2.13 ± 0.66), Fatigue/Energy (3.65 ± 1.08), and Sexual problems (3.78 ± 1.23). Because QLACS includes both positively and negatively framed domains, direct comparison of domain means should not be interpreted as a ranking of overall quality-of-life impairment.

The dispersion of responses was higher for Negative feelings (SD = 1.38), Cancer benefits (SD = 1.28), Family distress (SD = 1.26), and Positive feelings (SD = 1.25). The smallest standard deviations were observed for Financial problems (SD = 0.66), Pain (SD = 0.72), and Cognitive problems (SD = 0.79), indicating closer responses across participants for these domains.

The distributions were generally acceptable based on skewness and kurtosis values. A more pronounced deviation was observed for Pain, where the skewness was −1.498, and the kurtosis was 4.866, suggesting a clustering of responses toward the higher values of the scale (Table 4).

The normality of the distribution of scores on QLACS domains was verified by Kolmogorov–Smirnov and Shapiro–Wilk tests. For all 12 domains, the results were statistically significant (*p* < 0.001), indicating that the score distributions deviate from normality. The largest deviation from normal distribution was observed for the Pain domain (Shapiro–Wilk W = 0.722). Lower Shapiro–Wilk test values were also observed for Financial problems (W = 0.861), Family distress (W = 0.873), and Appearance concerns (W = 0.874). Based on these results, non-parametric tests were used for subsequent comparative analyses (Table 5).

### 3.4. Associations of QLACS Domain Scores with Age and Residence

Comparisons by residence were performed using the Mann–Whitney U test. For most QLACS domains, scores did not differ significantly between rural and urban participants. Significant differences were observed between the two domains. In the case of the domain Cognitive problems, urban participants had higher mean ranks than rural participants (120.97 vs. 98.91; U = 4765.500; *p* = 0.010), indicating higher scores in the urban group. The effect size was small (r = 0.17). For Family distress, the difference was more pronounced. Participants from rural areas had higher mean ranks than those from urban areas (135.17 vs. 98.37; U = 3984.000; *p* < 0.001), with an effect size close to the moderate level (r = 0.29). For the other domains, the differences between the two groups did not reach statistical significance (Table 6).

The association between age and QLACS scores was assessed using the Spearman coefficient. Of the 12 domains analyzed, age was significantly associated with Negative feelings, Sexual problems, and Recurrence distress.

For Negative feelings, a weak negative correlation was observed (rho = −0.200, *p* = 0.003), suggesting lower scores in older participants. Age was positively correlated with Sexual problems (rho = 0.296, *p* < 0.001). A weak positive association was also observed with Recurrence distress (rho = 0.135, *p* = 0.043); however, given the exploratory nature of these analyses and the absence of adjustment for multiple comparisons, this latter finding should be interpreted with caution. The most obvious association was with the Sexual problems domain, although its intensity was reduced to moderate (Table 7).

For the remaining QLACS domains, no significant correlations with age were identified.

Multivariable regression models including age and residential environment as a priori predictors were fitted for all 12 QLACS domains. Statistically significant overall models were observed for Cognitive problems, Family distress, Negative feelings, and Sexual problems. The remaining models were not statistically significant.

After adjusting for age, residential environment remained significantly associated with three domains. For Cognitive problems, urban participants had higher scores than rural participants, whereas for Family distress, rural participants had higher scores. For Sexual problems, residential environment was not significant in the unadjusted comparison but became significant after adjustment for age. This is not necessarily contradictory, because the adjusted model estimates the association with residence while controlling for age.

Age was significantly associated with Negative feelings and Sexual problems. Scores for Negative feelings decreased with age, while scores for Sexual problems increased. The model for Sexual problems explained the largest proportion of the variance in scores, R^2^ = 0.130, and the model for “Family distress” explained 10.6% of the variance. The models explained only a modest proportion of the variance in QLACS scores, with R^2^ values ranging from low to modest.

For “Recurrence distress”, the overall model was not statistically significant, although age had a positive association with scores in this domain. Therefore, this result should be interpreted with caution (Table 8). Overall, age and residential environment were associated with only selected QLACS dimensions. Residential environment seems more relevant for cognitive and family domains, while age is mainly associated with affective and sexual dimensions. VIF values were close to 1, indicating no multicollinearity problems between predictors.

### 3.5. Quality-of-Life Profiles Based on QLACS Domain Scores

Ward hierarchical classification was used as a preliminary step to determine the number of clusters based on the standardized scores of the QLACS domains. The resulting dendrogram is presented in Supplementary Figure S1. Based on this step, k-means analysis was performed for a three-cluster solution. The k-means analysis was performed on the standardized scores of the 12 QLACS domains and led to the identification of three groups of participants. Cluster 1 included 79 participants, Cluster 2 included 102 participants, and Cluster 3 included 43 participants, representing 35.3%, 45.5%, and 19.2% of the group.

Cluster 1 had higher scores for Negative feelings, Cognitive problems, Cancer benefits, and Pain, but lower scores for Social avoidance, Family distress, and Recurrence distress. Cluster 2 was characterized by higher values for Positive feelings, Social avoidance, Sexual problems, Financial problems, Family distress, Appearance concerns, and Recurrence distress, while scores for Negative feelings and Cancer benefits were below the group average. Cluster 3 had a different profile, with higher scores for Negative feelings and lower values for Positive feelings, Pain, Sexual problems, Financial problems, and Appearance concerns (Table 9). The analysis identified three exploratory profiles based on the standardized QLACS domain scores.

### 3.6. Differences Between QLACS Clusters

The comparisons between the three clusters were assessed using the Kruskal–Wallis test. To identify pairs of clusters with significant differences, post hoc comparisons with Bonferroni adjustment were subsequently applied. The results showed that differences between clusters were present on most QLACS domains. After Bonferroni correction, Cluster 1 and Cluster 2 differed significantly for most domains, except for Pain and Appearance concerns. Cluster 2 had higher scores for Positive feelings, Fatigue/Energy, Social avoidance, Sexual problems, Financial problems, Family distress, and Recurrence distress, while Cluster 1 had higher scores for Negative feelings, Cognitive problems, and Cancer benefits.

Between Cluster 1 and Cluster 3, the differences were significant for almost all domains, except for Fatigue/Energy and Recurrence distress. Cluster 1 had higher values for Positive feelings, Pain, Cognitive problems, Sexual problems, Financial problems, Cancer benefits, and Appearance concerns, and Cluster 3 had higher values for Social avoidance, Negative feelings, and Family distress.

In the comparison between Cluster 2 and Cluster 3, the differences remained significant for most domains, except for Social avoidance and Cognitive problems. Cluster 2 had higher scores for Positive feelings, Fatigue/Energy, “Pain, Sexual problems, Financial problems, Family distress, Appearance concerns and Recurrence distress, while Cluster 3 had higher values for Negative feelings and Cancer benefits (Table 10).

These results support the separation of the three QLACS profiles and show that the differences between the clusters have a distinct distribution across the domains of the questionnaire.

Age did not differ significantly between the three clusters, although participants in Cluster 2 had a higher mean rank compared to those in Cluster 1 and Cluster 3, H(2) = 5.752, *p* = 0.056.

The association between cluster and residential area was analyzed using the chi-square test. The result was statistically significant, χ^2^(2) = 7.921, *p* = 0.019, indicating that the distribution of rural and urban participants was not identical across the three clusters. In Cluster 1, urban participants predominated, 58 out of 79 cases (73.4%). In Cluster 2, the distribution was more balanced, with 48 rural participants (47.1%) and 54 urban participants (52.9%). Cluster 3 had a structure close to the general distribution of the group, with 17 participants from rural areas (39.5%) and 26 from urban areas (60.5%) (Table 11).

While age did not differ significantly between the three clusters, the environment of residence was significantly associated with cluster membership, χ^2^(2) = 7.921, *p* = 0.019, Cramer’s V = 0.188.

## 4. Discussion

In the present study, the Romanian QLACS showed good internal consistency across all 12 domains. These findings were broadly comparable with those reported in previous psychometric evaluations of the QLACS [5,6,24,37]. Previous studies have also examined construct validity, temporal stability, and factorial structure in different survivor populations. In contrast, the present study primarily provides evidence on internal consistency and exploratory structural characteristics, while test–retest reliability, convergent and divergent validity, and confirmatory factor analysis were not assessed.

Principal component analysis identified an 11-component solution, which did not fully reproduce the theoretical 12-domain structure of the QLACS. The difference was mainly related to the Cognitive problems domain, which was not as clearly separated from Recurrence distress. Some of the cognitive items were grouped together with items related to the fear of recurrence. However, reported Cognitive problems after cancer are varied and can be influenced by fatigue, pain, sleep disturbances, anxiety, and emotional distress [5,6,7]. At the same time, persistent worries and hypervigilance associated with fear of recurrence can impair concentration and attention, which may partly explain the closeness between the two domains [9,10,11]. Although the 11 components explained a high proportion of the total variance, this exploratory component solution should be interpreted with caution and should not be considered confirmation of the theoretical 12-domain structure. The number of components was established based on the Kaiser criterion, a method that can sometimes overestimate the number of factors. In a subsequent study, the structure should be verified by parallel analysis and confirmatory factor analysis on an independent group. In a subsequent study, the structure should be verified by parallel analysis and confirmatory factor analysis on an independent group. Differences from the original model are not uncommon in cultural adaptation studies, especially when some dimensions are clinically or psychologically close [5,7,24,37].

Relatively high mean scores were observed for Cognitive problems, Appearance concerns, Pain, and Recurrence distress. These domain scores should be interpreted individually rather than as a direct ranking of impairment across QLACS dimensions. Cognitive difficulties and pain can persist after cancer treatment and can affect daily activities, autonomy, and social participation [9,10,12]. In the case of women, bodily changes caused by the disease and treatment can influence body perception, self-confidence and intimate life [12,13,14,15].

The high score for Cancer benefits shows that some participants also identified positive changes after the cancer experience, such as reassessing priorities or developing better coping strategies. However, these changes do not exclude the presence of difficulties. In the same group, Cognitive problems, pain, and fear of recurrence also had an important weight, which shows that positive and negative aspects can coexist.

Lower scores for Financial problems, Fatigue/Energy and Sexual problems do not mean that these areas are irrelevant. Their intensity may vary depending on the type of cancer, treatment followed, age, professional status, income, access to services, and time since diagnosis [13,21,22]. In the present study, sexual problems increased with age, which supports the inclusion of sexual health in the routine assessment of oncology patients, even if this topic is still insufficiently addressed in practice [15,21].

Residential environment was associated with Cognitive problems and Family distress. Participants from rural areas had higher scores for Family distress. This result may be related to more difficult access to services, longer distances to health centers, indirect costs, and greater dependence on family support [21,22,23]. The association remained after adjusting for age, suggesting that the difference is not explained solely by the age structure of the two groups.

Higher cognitive scores in urban areas are more difficult to interpret. They may reflect differences in education, professional activity, access to information, or symptom reporting. However, these explanations remain hypothetical, as these variables were not included in the analysis. Age was associated with Negative feelings, Sexual problems, and Recurrence distress. Older participants reported lower scores for negative affectivity, possibly due to different coping mechanisms or a different way of expressing distress. In contrast, sexual problems increased with age, a finding consistent with the role of hormonal changes, comorbidities, cancer treatments, and body image [13,14,15]. The association with Recurrence distress was significant in the Spearman analysis, but the regression model was not significant overall, so this result should be interpreted with caution.

Because these analyses involved multiple QLACS domains and were not adjusted for multiple testing, they should be regarded as exploratory. Accordingly, findings with *p*-values close to 0.05, particularly the weak association between age and Recurrence distress, require cautious interpretation and confirmation in independent samples.

Cluster analysis identified three exploratory quality-of-life profiles within the present sample, characterized by different combinations of emotional, cognitive, family, financial, sexual, and recurrence-related concerns. Similar heterogeneity has been reported in previous cluster and latent profile studies of cancer survivors [29,30,31,32,33,34,38]. However, because cluster stability and internal validation were not assessed, these profiles should not be regarded as validated or clinically established subgroups. The observed profiles may be useful for generating hypotheses regarding differences in supportive-care needs, but their clinical relevance requires confirmation in independent samples.

Age did not differ significantly between the three clusters. Although residential environment was significantly associated with cluster membership, the effect size was small (Cramer’s V = 0.188), indicating limited practical strength of this association. Residency therefore explains only a small part of the distribution of profiles, and differences between participants appear to depend on a broader combination of clinical, psychological, and social factors [39,40,41].

### Study Limitations

The study has several limitations that must be taken into account when interpreting the results. The group was composed exclusively of women, so the conclusions cannot be automatically extended to the male population. In addition, the cross-sectional nature of the research does not allow the establishment of causal relationships between age, residential environment, and QLACS scores.

The translation procedure was also limited by the absence of back-translation, independent bilingual expert review, an expert committee, and formal content validity assessment. Although comprehensibility was checked in the first 100 participants, this does not constitute a complete evaluation of linguistic and content equivalence.

The structural analysis was exploratory and was based on principal component analysis and the Kaiser eigenvalue >1 criterion. More robust approaches, such as exploratory factor analysis combined with parallel analysis and examination of the scree plot, were not applied. The identified structure should therefore be confirmed using confirmatory factor analysis in an independent sample.

The sample size was also relatively modest for a psychometric analysis involving 47 questionnaire items, and the structural findings should therefore be interpreted cautiously.

Internal consistency was assessed using Cronbach’s alpha only. Although the coefficients were high, alpha does not by itself demonstrate unidimensionality or construct validity and may also be influenced by item redundancy. Additional reliability indices, such as McDonald’s omega, should be considered in future psychometric evaluations.

Test–retest reliability was not assessed. In addition, convergent, discriminant/divergent, and criterion-related validity were not examined, so the present study does not provide a comprehensive psychometric validation of the Romanian QLACS.

The cluster analysis was exploratory. The three-cluster solution was selected based on inspection of the dendrogram and agglomeration process, without additional internal validation using methods such as silhouette coefficients, bootstrap stability analysis, alternative initializations, or replication in an independent sample. Therefore, the identified clusters should be interpreted as sample-specific exploratory profiles rather than validated clinical subgroups.

Another important limitation is the absence of detailed clinical information, including breast cancer stage, treatment modalities, current treatment status, time since diagnosis, time since treatment completion, recurrence or metastatic status, and comorbidities. These characteristics may influence several QLACS domains, including Cognitive problems, pain, fatigue, sexual functioning, appearance concerns, and Recurrence distress. Their absence limits the interpretation of both the descriptive findings and the psychometric results and prevents a more detailed assessment of whether QLACS performance differs across clinical subgroups.

Although Bonferroni correction was applied to post hoc comparisons between clusters, no multiplicity adjustment was applied to the analyses of associations between the 12 QLACS domains, age, and residential environment or to the regression analyses. These analyses were exploratory, and the possibility of type I error cannot be excluded, particularly for associations with *p*-values close to 0.05.

Participants were recruited by convenience sampling from a single hospital, which limits the generalizability of the findings. Information on the number of eligible patients approached, refusals, incomplete questionnaires, and the overall response rate was not systematically recorded, limiting the assessment of possible selection and non-response bias.

## 5. Conclusions

The Romanian QLACS showed good internal consistency and preliminary exploratory structural evidence in women with breast cancer. The 11-component solution broadly reflected the multidimensional structure of the instrument but did not fully reproduce the theoretical 12-domain model, mainly because the Cognitive problems domain did not emerge as a distinct component and partially overlapped with Recurrence distress.

Age and residential environment were associated with selected QLACS domains, although these findings should be interpreted as exploratory. The three quality-of-life profiles identified by cluster analysis should likewise be considered exploratory and sample-specific rather than validated clinical subgroups. The findings provide preliminary evidence regarding the psychometric performance of the Romanian QLACS in women with breast cancer.

Further studies in independent samples are needed to confirm the psychometric properties of the Romanian QLACS, including confirmatory factor analysis, test–retest reliability, convergent and divergent validity, and assessment of cluster stability. Studies including more detailed clinical characteristics would also help determine whether the instrument performs consistently across different breast cancer subgroups.

## Figures and Tables

**Table 1:** Characteristics of the studied group.

Characteristic	Result
Number of participants	224
Female sex	224 (100%)
Breast cancer diagnosis	224 (100%)
Age	
Age, mean ± SD	50.84 ± 14.21 years
Age, median	52 years
Age, minimum–maximum	23–81 years
Residence	
Rural residence	86 (38.4%)
Urban residence	138 (61.6%)

**Table 2:** Internal consistency reliability indices of the questionnaire domains.

	Domain	Items	Cronbach’s Alpha	No. of Items
Domain 1.	Positive feelings	6, 8, 22, 28	0.899	4
Domain 2.	Fatigue/Energy	1, 5, 11, 14	0.872	4
Domain 3.	Social avoidance	15, 18, 20, 25	0.852	4
Domain 4.	Pain	13, 17, 21, 27	0.866	4
Domain 5.	Negative feelings	7, 9, 19, 24	0.925	4
Domain 6.	Cognitive problems	2, 3, 4, 23	0.832	4
Domain 7.	Sexual problems	10, 12, 16, 26	0.857	4
Domain 8.	Financial problems	30, 37, 43, 45	0.870	4
Domain 9.	Cancer benefits	29, 32, 40, 41	0.893	4
Domain 10.	Family distress	31, 34, 42	0.855	3
Domain 11.	Appearance concerns	33, 35, 38, 44	0.880	4
Domain 12.	Recurrence distress	36, 39, 46, 47	0.884	4

**Table 3:** Kaiser–Meyer–Olkin measure and Bartlett’s test of sphericity.

Kaiser–Meyer–Olkin Measure of Sampling Adequacy.		0.877
Bartlett’s Test of Sphericity	Approx. Chi-Square	6859.669
	df	1081
	Sig.	*p* < 0.001

**Table 4:** Descriptive statistics of QLACS domain scores.

Domain	Mean ± SD	Median	Minimum–Maximum	Skewness	Kurtosis
Positive feelings	4.64 ± 1.25	5.00	2.50–7.00	0.020	−1.185
Fatigue/Energy	3.65 ± 1.08	3.50	1.50–6.00	0.048	−0.868
Social avoidance	4.69 ± 1.18	5.00	2.00–7.00	−0.252	−0.143
Pain	5.29 ± 0.72	5.00	2.00–6.00	−1.498	4.866
Negative feelings	4.33 ± 1.38	4.75	1.75–6.00	−0.562	−1.076
Cognitive problems	5.92 ± 0.79	6.00	3.00–7.00	−0.266	−0.242
Sexual problems	3.78 ± 1.23	3.25	2.00–6.00	0.468	−0.819
Financial problems	2.13 ± 0.66	2.00	1.00–3.00	−0.297	−0.801
Cancer benefits	5.11 ± 1.28	5.00	3.00–7.00	−0.323	−1.011
Family distress	5.00 ± 1.26	5.00	2.00–7.00	−0.823	−0.095
Appearance concerns	5.57 ± 1.19	6.00	2.00–7.00	−0.924	1.103
Recurrence distress	5.13 ± 1.03	5.00	3.00–7.00	−0.427	−0.203

**Table 5:** Tests of normality for QLACS domain scores.

Domain	Kolmogorov–Smirnov			Shapiro–Wilk		
	Statistic	df	*p*-Value	Statistic	df	*p*-Value
Positive feelings	0.159	224	<0.001	0.925	224	<0.001
Fatigue/Energy	0.137	224	<0.001	0.959	224	<0.001
Social avoidance	0.187	224	<0.001	0.948	224	<0.001
Pain	0.265	224	<0.001	0.722	224	<0.001
Negative feelings	0.165	224	<0.001	0.885	224	<0.001
Cognitive problems	0.202	224	<0.001	0.881	224	<0.001
Sexual problems	0.236	224	<0.001	0.884	224	<0.001
Financial problems	0.227	224	<0.001	0.861	224	<0.001
Cancer benefits	0.221	224	<0.001	0.893	224	<0.001
Family distress	0.224	224	<0.001	0.873	224	<0.001
Appearance concerns	0.177	224	<0.001	0.874	224	<0.001
Recurrence distress	0.210	224	<0.001	0.902	224	<0.001

Lilliefors significance correction was applied for the Kolmogorov–Smirnov test. Values reported as *p* < 0.001 correspond to SPSS 26.0 Sig. = 0.000. df = degrees of freedom.

**Table 6:** Comparison of QLACS domain scores according to residence (Mann–Whitney U test).

Domain	Rural Mean Rank (*n* = 86)	Urban Mean Rank (*n* = 138)	Mann–Whitney U	Z	*p*-Value	Effect Size r
Positive feelings	111.99	112.82	5890.000	−0.094	0.925	0.01
Fatigue/Energy	104.99	117.18	5288.500	−1.378	0.168	0.09
Social avoidance	117.58	109.34	5497.500	−0.946	0.344	0.06
Pain	112,72	112.37	5915.500	−0.044	0.965	0.00
Negative feelings	102.78	118.55	5098.500	−1.778	0.075	0.12
Cognitive problems	98.91	120.97	4765.500	−2.592	0.010	0.17
Sexual problems	103.71	117.98	5178.000	−1.659	0.097	0.11
Financial problems	109.12	114.61	5643.000	−0.647	0.517	0.04
Cancer benefits	116.44	110.05	5595.500	−0.738	0.460	0.05
Family distress	135.17	98.37	3984.000	−4.299	<0.001	0.29
Appearance concerns	106.79	116.06	5443.000	−1.075	0.282	0.07
Recurrence distress	111.80	112.93	5874.000	−0.133	0.894	0.01

Effect size was calculated as r = |Z|/√N. *p*-values are two-tailed. Residence groups: rural (*n* = 86) and urban (*n* = 138).

**Table 7:** Spearman correlations between age and QLACS domain scores.

Domain	Spearman’s Rho	*p*-Value
Positive feelings	0.086	0.201
Fatigue/Energy	0.035	0.605
Social avoidance	−0.024	0.722
Pain	0.073	0.274
Negative feelings	−0.200	0.003
Cognitive problems	0.107	0.110
Sexual problems	0.296	<0.001
Financial problems	−0.047	0.482
Cancer benefits	0.060	0.371
Family distress	0.095	0.155
Appearance concerns	−0.011	0.875
Recurrence distress	0.135	0.043

**Table 8:** Multivariable linear regression models for all QLACS domains.

Outcome	Model Fit	Predictor	B	SE	β	95% CI for B	*p*-Value	VIF
Positive feelings	F(2,221) = 0.598; *p* = 0.551; R^2^ = 0.005; Adj. R^2^ = −0.004	Residence	0.015	0.175	0.006	−0.329–0.360	0.930	1.038
		Age	0.007	0.006	0.074	−0.005–0.018	0.278	1.038
Fatigue/Energy	F(2,221) = 1.875; *p* = 0.156; R^2^ = 0.017; Adj. R^2^ = 0.008	Residence	0.283	0.151	0.128	−0.014–0.580	0.061	1.038
		Age	0.004	0.005	0.055	−0.006–0.014	0.418	1.038
Social avoidance	F(2,221) = 1.102; *p* = 0.334; R^2^ = 0.010; Adj. R^2^ = 0.001	Residence	−0.238	0.165	−0.098	−0.563–0.088	0.151	1.038
		Age	−0.004	0.006	−0.043	−0.015–0.008	0.531	1.038
Pain	F(2,221) = 0.092; *p* = 0.913; R^2^ = 0.001; Adj. R^2^ = −0.008	Residence	−0.003	0.101	−0.002	−0.202–0.195	0.972	1.038
		Age	0.001	0.003	0.028	−0.005–0.008	0.681	1.038
Negative feelings	F(2,221) = 6.442; *p* = 0.002; R^2^ = 0.055; Adj. R^2^ = 0.047	Residence	0.234	0.188	0.083	−0.137–0.605	0.216	1.038
		Age	−0.020	0.006	−0.204	−0.033–−0.007	0.002	1.038
Cognitive problems	F(2,221) = 4.432; *p* = 0.013; R^2^ = 0.039; Adj. R^2^ = 0.030	Residence	0.293	0.109	0.181	0.079–0.507	0.007	1.038
		Age	0.007	0.004	0.117	−0.001–0.014	0.082	1.038
Sexual problems	F(2,221) = 16.470; *p* < 0.001; R^2^ = 0.130; Adj. R^2^ = 0.122	Residence	0.385	0.162	0.152	0.066–0.704	0.018	1.038
		Age	0.031	0.006	0.357	0.020–0.042	<0.001	1.038
Financial problems	F(2,221) = 0.212; *p* = 0.810; R^2^ = 0.002; Adj. R^2^ = −0.007	Residence	0.041	0.093	0.030	−0.142–0.224	0.658	1.038
		Age	−0.001	0.003	−0.026	−0.007–0.005	0.703	1.038
Cancer benefits	F(2,221) = 0.245; *p* = 0.783; R^2^ = 0.002; Adj. R^2^ = −0.007	Residence	−0.112	0.180	−0.042	−0.467–0.244	0.536	1.038
		Age	0.001	0.006	0.014	−0.011–0.013	0.840	1.038
Family distress	F(2,221) = 13.089; *p* < 0.001; R^2^ = 0.106; Adj. R^2^ = 0.098	Residence	−0.829	0.167	−0.321	−1.158–−0.499	<0.001	1.038
		Age	0.002	0.006	0.019	−0.010–0.013	0.768	1.038
Appearance concerns	F(2,221) = 0.333; *p* = 0.717; R^2^ = 0.003; Adj. R^2^ = −0.006	Residence	0.134	0.167	0.055	−0.196–0.463	0.425	1.038
		Age	−0. 00008	0.006	−0.001	−0.011–0.011	0.989	1.038
Recurrence distress	F(2,221) = 2.500; *p* = 0.084; R^2^ = 0.022; Adj. R^2^ = 0.013	Residence	0.102	0.143	0.048	−0.180–0.383	0.478	1.038
		Age	0.011	0.005	0.150	0.001–0.021	0.028	1.038

**Table 9:** Quality-of-life profiles based on standardized QLACS domain scores.

QLACS Domain	Cluster 1 (*n* = 79; 35.3%)	Cluster 2 (*n* = 102; 45.5%)	Cluster 3 (*n* = 43; 19.2%)
Positive feelings	−0.015	0.417	−0.963
Fatigue/Energy	−0.181	0.219	−0.186
Social avoidance	−0.663	0.396	0.279
Pain	0.312	0.040	−0.669
Negative feelings	0.409	−0.619	0.715
Cognitive problems	0.363	−0.151	−0.308
Sexual problems	−0.132	0.344	−0.573
Financial problems	−0.161	0.422	−0.705
Cancer benefits	0.498	−0.431	0.107
Family distress	−0.801	0.575	0.109
Appearance concerns	0.192	0.358	−1.203
Recurrence distress	−0.324	0.404	−0.362

Values represent standardized QLACS domain scores. K-means clustering was performed using the 12 standardized domain scores and converged after 12 iterations.

**Table 10:** Bonferroni-adjusted post hoc pairwise comparisons between QLACS clusters.

Domain	C1 vs. C2	C1 vs. C3	C2 vs. C3
Positive feelings	C2 > C1, *p*_adj = 0.009	C1 > C3, *p*_adj < 0.001	C2 > C3, *p*_adj < 0.001
Fatigue/Energy	C2 > C1, *p*_adj = 0.033	ns	C2 > C3, *p*_adj = 0.048
Social avoidance	C2 > C1, *p*_adj < 0.001	C3 > C1, *p*_adj < 0.001	ns
Pain	ns	C1 > C3, *p*_adj < 0.001	C2 > C3, *p*_adj = 0.009
Negative feelings	C1 > C2, *p*_adj < 0.001	C3 > C1, *p*_adj = 0.030	C3 > C2, *p*_adj < 0.001
Cognitive problems	C1 > C2, *p*_adj = 0.003	C1 > C3, *p*_adj < 0.001	ns
Sexual problems	C2 > C1, *p*_adj = 0.009	C1 > C3, *p*_adj < 0.001	C2 > C3, *p*_adj < 0.001
Financial problems	C2 > C1, *p*_adj < 0.001	C1 > C3, *p*_adj = 0.012	C2 > C3, *p*_adj < 0.001
Cancer benefits	C1 > C2, *p*_adj < 0.001	C1 > C3, *p*_adj = 0.012	C3 > C2, *p*_adj = 0.009
Family distress	C2 > C1, *p*_adj < 0.001	C3 > C1, *p*_adj < 0.001	C2 > C3, *p*_adj < 0.001
Appearance concerns	ns	C1 > C3, *p*_adj < 0.001	C2 > C3, *p*_adj < 0.001
Recurrence distress	C2 > C1, *p*_adj < 0.001	ns	C2 > C3, *p*_adj < 0.001

ns = not significant after Bonferroni correction.

**Table 11:** Distribution of residence according to QLACS cluster membership.

Cluster	Rural *n* (%)	Urban *n* (%)	Total
Cluster 1	21 (26.6%)	58 (73.4%)	79
Cluster 2	48 (47.1%)	54 (52.9%)	102
Cluster 3	17 (39.5%)	26 (60.5%)	43
Total	86 (38.4%)	138 (61.6%)	224

χ^2^(2) = 7.921, *p* = 0.019; Cramer’s V = 0.188. Percentages are calculated within clusters.

## Data Availability

The original contributions presented in this study are included in the article. Further inquiries can be directed to the corresponding author.

## Supplementary Materials

The following supporting information can be downloaded at: https://www.mdpi.com/article/10.3390/jcm15187151/s1, Figure S1: Dendrogram obtained using Ward’s method; Table S1: Item-level descriptive statistics and Cronbach’s Alpha if Item Deleted; Table S2: Communalities of questionnaire items after principal component extraction; Table S3: Total variance explained by the extracted components; Table S4: Pattern matrix of the principal component analysis after Oblimin rotation.

## Abbreviations

The following abbreviations are used in this manuscript:

QLACS	Quality of Life in Adult Cancer Survivors
OECD	Organisation for Economic Co-operation and Development
EORTC QLQ-C30	European Organisation for Research and Treatment of Cancer
FACT-B	Functional Assessment of Cancer Therapy–Breast
SF-36	Short Form-36 Health Survey
Z	z-scoreR
R	Coefficient of determination
*p*	*p*-value
SD	Standard Deviation
A	Cronbach’s Alpha
W	Shapiro–Wilk
U	Urban
R	Rural
R	Effect size
Rho	Coeficientul de corelație Spearman
B	Unstandardized regression coefficient
SE	Standard error
B	Standardized regression coefficient
95% CI	95% Confidence interval
VIF	Variance Inflation Factor
C	Cluster
χ^2^	Chi-square
