# Supplementary material for: Psychometric Evaluation of the Romanian Quality of Life in Adult Cancer Survivors (QLACS) in Women with Breast Cancer: A Cross-Sectional Study
Source: J Clin Med. 2026 Sep 15;15(18):7151. doi: 10.3390/jcm15187151 (PMC13607059; doi:10.3390/jcm15187151)

Supplementary Table S1. Item-level descriptive statistics and Cronbach's Alpha if Item Deleted

		Mean	Std. Deviation	Cronbach's Alpha if Item Deleted
Positive feelings	You felt happy.	4,94	1,387	0,837
	You enjoyed life	4,71	1,411	0.871
	You had a positive outlook on life.	4,42	1,443	0.882
	You were content with your life.	4,49	1,452	0.888
Fatigue/Energy	You had the energy to do the things you wanted to do.	3,83	1,234	,805
	You felt fatigued.	3,45	1,277	,854
	You did not have energy to do the things you wanted to do.	3,65	1,283	,850
	You felt tired a lot.	3,67	1,288	,833
Social avoidance	You were reluctant to start new relationships.	5,00	1,286	,810
	You avoided social gatherings.	4,42	1,513	,834
	You avoided your friends.	4,62	1,450	,798
	You were reluctant to meet new people.	4,74	1,410	,807
Pain evaluates	You were bothered by pain that kept you from doing the things you wanted to do.	5,35	,742	,825
	Your mood was disrupted by pain or its treatment.	5,24	,940	,853
	You had aches or pains.	5,27	,888	,823
	Pain or its treatment interfered with your social activities.	5,31	,804	,816
Negative feelings	You felt blue or depressed	4,22	1,443	,913
	You worried about little things.	4,50	1,506	,895
	You were bothered by mood swings.	4,30	1,549	,906
	You felt anxious.	4,29	1,600	,895
Cognitive problems	You had difficulty doing activities that require concentrating.	6,00	,855	,774
	You were bothered by having a short attention span	5,79	1,123	,825
	You had trouble remembering things.	5,83	1,076	,785

	You were bothered by forgetting what you started to do.	6,08	,765	,773
Sexual problems	You were bothered by being unable to function sexually.	3,91	1,483	,781
	You were dissatisfied with your sex life.	3,74	1,481	,819
	You lacked interest in sex.	3,69	1,470	,825
	You avoided sexual activity.	3,77	1,467	,842
Financial problems	You had financial problems because of the cost of cancer surgery or treatment.	2,18	,800	,844
	You had problems with insurance because of cancer.	2,10	,771	,831
	You had money problems that arose because you had cancer.	2,15	,798	,838
	You had financial problems due to a loss of income as a result of cancer	2,08	,744	,821
Cancer benefits	You appreciated life more because of having had cancer.	5,16	1,433	,844
	You realized that having had cancer helps you cope better with problems now.	5,05	1,514	,871
	You felt that cancer helped you to recognize what is important in life.	5,09	1,490	,854
	You felt better able to deal with stress because of having had cancer.	5,12	1,461	,881
Family distress	You worried that your family members were at risk of getting cancer.	5,09	1,344	,796
	You worried about whether your family members might have cancer-causing genes.	4,90	1,519	,810
	You worried about whether your family members should have genetic tests for cancer.	5,02	1,417	,785
Appearance concerns	You were self-conscious about the way you look because of your cancer or its treatment.	5,63	1,295	,860
	You felt unattractive because of your cancer or its treatment.	5,54	1,442	,851

	You were bothered by hair loss from cancer treatment.	5,60	1,362	,818
	You felt people treated you differently because of changes to your appearance due to your cancer or its treatment.	5,51	1,455	,854
Recurrence distress	You worried about dying from cancer.	5,08	1,226	,860
	You worried about cancer coming back.	5,08	1,252	,853
	Whenever you felt a pain, you worried that it might be cancer again.	5,16	1,163	,837
	You were preoccupied with concerns about cancer.	5,18	1,124	,855

Supplementary Table S2. Communalities of questionnaire items after principal component extraction

	Initial	After extraction
You had the energy to do the things you wanted to do.	,808	,823
You felt fatigued.	,686	,695
You did not have energy to do the things you wanted to do.	,667	,683
You felt tired a lot.	,718	,730
You had difficulty doing activities that require concentrating.	,712	,771
You were bothered by having a short attention span	,595	-
You had trouble remembering things.	,640	,-
You were bothered by forgetting what you started to do.	,796	,828
You felt happy.	,873	,878
You enjoyed life	,773	,775
You had a positive outlook on life.	,761	,768
You were content with your life.	,735	,737
You felt blue or depressed	,783	,789
You worried about little things.	,846	,868
You were bothered by mood swings.	,806	,820
You felt anxious.	,849	,857
You were bothered by being unable to function sexually.	,799	,791
You were dissatisfied with your sex life.	,701	,748
You lacked interest in sex.	,713	,764
You avoided sexual activity.	,635	-
You were bothered by pain that kept you from doing the things you wanted to do.	,747	,767
Your mood was disrupted by pain or its treatment.	,664	-
You had aches or pains.	,750	,818
Pain or its treatment interfered with your social activities.	,748	,774
You were reluctant to start new relationships.	,763	,800
You avoided social gatherings.	,683	-
You avoided your friends.	,744	,803
You were reluctant to meet new people.	,713	,744
You appreciated life more because of having had cancer.	,815	,804

You realized that having had cancer helps you cope better with problems now.	,730	,774
You felt that cancer helped you to recognize what is important in life.	,767	,790
You felt better able to deal with stress because of having had cancer.	,696	-
You had financial problems because of the cost of cancer surgery or treatment.	,676	-
You had problems with insurance because of cancer.	,700	,690
You had money problems that arose because you had cancer.	,736	,787
You had financial problems due to a loss of income as a result of cancer	,754	,765
You worried that your family members were at risk of getting cancer.	,739	,761
You worried about whether your family members might have cancer-causing genes.	,722	,774
You worried about whether your family members should have genetic tests for cancer.	,768	,792
You were self-conscious about the way you look because of your cancer or its treatment.	,713	,718
You felt unattractive because of your cancer or its treatment.	,746	,746
You were bothered by hair loss from cancer treatment.	,816	,817
You felt people treated you differently because of changes to your appearance due to your cancer or its treatment.	,719	,727
You worried about dying from cancer.	,686	-
You worried about cancer coming back.	,720	,802
Whenever you felt a pain, you worried that it might be cancer again.	,751	,800
You were preoccupied with concerns about cancer.	,692	-

Supplementary Table S3. Total variance explained by the extracted components

Component	Initial Eigenvalues			Extraction Sums of Squared Loadings			Rotation Sums of Squared Loadings[a]
	Total	% of Variance	Cumulative %	Total	% of Variance	Cumulative %	Total
1	7,079	15,062	15,062	7,079	15,062	15,062	4,534
2	5,009	10,657	25,719	5,009	10,657	25,719	3,845
3	3,991	8,492	34,212	3,991	8,492	34,212	3,332
4	3,530	7,510	41,722	3,530	7,510	41,722	4,116
5	3,200	6,808	48,529	3,200	6,808	48,529	3,067
6	2,631	5,597	54,126	2,631	5,597	54,126	3,372
7	2,376	5,055	59,182	2,376	5,055	59,182	3,341
8	2,050	4,362	63,543	2,050	4,362	63,543	3,537
9	1,829	3,892	67,436	1,829	3,892	67,436	4,258
10	1,675	3,563	70,999	1,675	3,563	70,999	4,267
11	1,287	2,739	73,738	1,287	2,739	73,738	3,802
12	0,859	1,827	75,565				
13	0,690	1,469	77,034				
14	0,641	1,365	78,398				
15	0,605	1,286	79,685				
16	0,591	1,257	80,942				
17	0,543	1,156	82,097				
18	0,523	1,112	83,209				
19	0,519	1,105	84,314				
20	0,489	1,041	85,355				
21	0,464	0,988	86,343				
22	0,453	0,964	87,307				
23	0,428	0,912	88,219				
24	0,424	0,903	89,122				
25	0,376	0,799	89,921				
26	0,369	0,785	90,706				
27	0,353	0,751	91,457				
28	0,321	0,683	92,140				
29	0,312	0,664	92,804				
30	0,292	0,622	93,426				
31	0,288	0,613	94,038				
32	0,270	0,575	94,613				
33	0,254	0,540	95,153				
34	0,242	0,515	95,668				
35	0,225	0,478	96,146				
36	0,219	0,466	96,612				
37	0,205	0,435	97,047				
38	0,192	0,408	97,455				
39	0,177	0,376	97,832				
40	0,175	0,371	98,203				
41	0,160	0,340	98,543				
42	0,150	0,318	98,862				

43	0,141	0,300	99,162	
44	0,121	0,257	99,418	
45	0,103	0,219	99,637	
46	0,090	0,192	99,829	
47	0,080	0,171	100,000	

Extraction Method: Principal Component Analysis.
When components are correlated, sums of squared loadings cannot be added to obtain a total variance

Supplementary Table S4. Pattern matrix of the principal component analysis after Oblimin rotation

**Item**	**1**	**2**	**3**	**4**	**5**	**6**	**7**	**8**	**9**	**10**	**11**
You felt anxious.	-,872										
You were bothered by mood swings.	-,853										
You worried about little things.	-,845										
You felt blue or depressed	-,833										
You worried about dying from cancer.		-,819									
You worried about cancer coming back.		-,794									
Whenever you felt a pain, you worried that it might be cancer again.		-,771									
You were preoccupied with concerns about cancer.		-,754									
You were bothered by forgetting what you started to do.		,446									
You were bothered by having a short attention span		,433									
You had difficulty doing activities that require concentrating.		,402									
You were bothered by pain that kept you from doing the things you wanted to do.			,859								
Your mood was disrupted by pain or its treatment.			,849								
You had aches or pains.			,826								
Pain or its treatment interfered with your social activities.			,800								
You appreciated life more because of having had cancer.				,895							
You realized that having had cancer helps you cope better with problems now.				,863							
You felt that cancer helped you to recognize what is important in life.				,853							
You felt better able to deal with stress because of having had cancer.				,799							
You worried that your family members were at risk of getting cancer.					-,848						

Item	1	2	3	4	5	6	7	8	9	10	11
You worried about whether your family members might have cancer-causing genes.					-,821						
You worried about whether your family members should have genetic tests for cancer.					-,799						
You had trouble remembering things.											
You were reluctant to start new relationships.						,871					
You avoided social gatherings.						,830					
You avoided your friends.						,818					
You were reluctant to meet new people.						,805					
You had the energy to do the things you wanted to do.							,841				
You felt fatigued.							,801				
You did not have energy to do the things you wanted to do.							,794				
You felt tired a lot.							,760				
You were self-conscious about the way you look because of your cancer or its treatment.								,884			
You felt unattractive because of your cancer or its treatment.								,848			
You were bothered by hair loss from cancer treatment.								,827			
You felt people treated you differently because of changes to your appearance due to your cancer or its treatment.								,818			
You had a positive outlook on life.									,883		
You were content with your life.									,860		
You felt happy.									,845		
You enjoyed life									,823		
You had financial problems because of the cost of cancer surgery or treatment.										-,892	
You had problems with insurance because of cancer.										-,824	
You had money problems that arose because you had cancer.										-,813	

Item	1	2	3	4	5	6	7	8	9	10	11
You had financial problems due to a loss of income as a result of cancer										-,764	
You were bothered by being unable to function sexually.											,852
You were dissatisfied with your sex life.											,842
You lacked interest in sex.											,818
You avoided sexual activity.											,750

Extraction Method: Principal Component Analysis. Rotation Method: Oblimin with Kaiser Normalization. Rotation converged in 22 iterations. Only loadings ≥ 0.40 are displayed.

Supplementary Figure S1. Dendrogram obtained using Ward's method

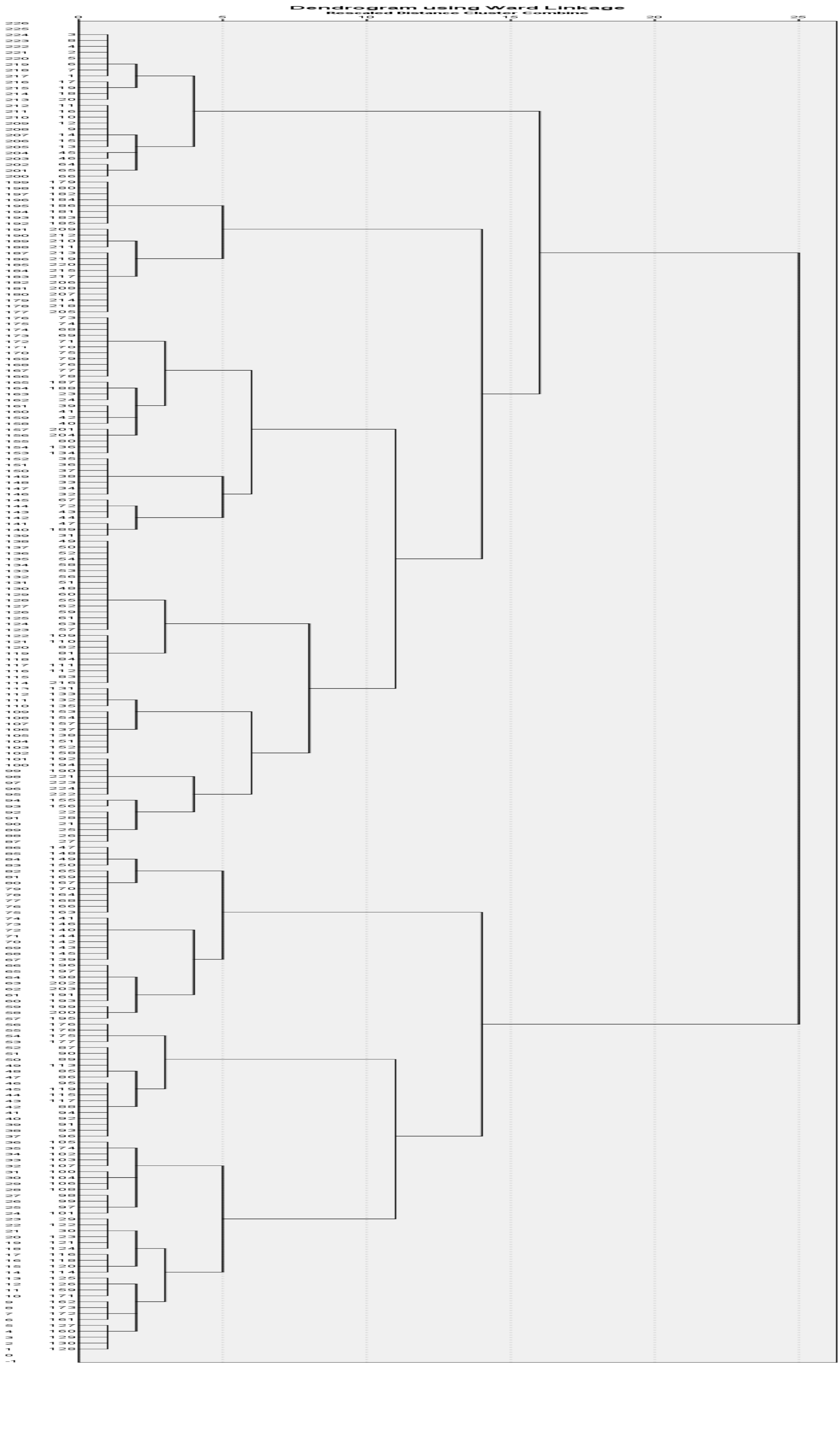

Supplement: Supplementary file 1 [file jcm-15-07151-s001.zip › jcm-4478381-supplementary.pdf]
